# Recent and Current Advances in FDG-PET Imaging within the Field of Clinical Oncology in NSCLC: A Review of the Literature

**DOI:** 10.3390/diagnostics10080561

**Published:** 2020-08-05

**Authors:** Kaoru Kaseda

**Affiliations:** Department of Thoracic Surgery, Sagamihara Kyodo Hospital, Kanagawa 252-5188, Japan; kaseda@wb4.so-net.ne.jp; Tel.: +81-42-772-4291; Fax: +81-42-771-6709

**Keywords:** non-small-cell lung cancer, FDG-PET/CT, metastasis, surgery

## Abstract

Lung cancer is the leading cause of cancer-related deaths around the world, the most common type of which is non-small-cell lung cancer (NSCLC). Computed tomography (CT) is required for patients with NSCLC, but often involves diagnostic issues and large intra- and interobserver variability. The anatomic data obtained using CT can be supplemented by the metabolic data obtained using fluorodeoxyglucose F 18 (FDG) positron emission tomography (PET); therefore, the use of FDG-PET/CT for staging NSCLC is recommended, as it provides more accuracy than either modality alone. Furthermore, FDG-PET/magnetic resonance imaging (MRI) provides useful information on metabolic activity and tumor cellularity, and has become increasingly popular. A number of studies have described FDG-PET/MRI as having a high diagnostic performance in NSCLC staging. Therefore, multidimensional functional imaging using FDG-PET/MRI is promising for evaluating the activity of the intratumoral environment. Radiomics is the quantitative extraction of imaging features from medical scans. The chief advantages of FDG-PET/CT radiomics are the ability to capture information beyond the capabilities of the human eye, non-invasiveness, the (virtually) real-time response, and full-field analysis of the lesion. This review summarizes the recent advances in FDG-PET imaging within the field of clinical oncology in NSCLC, with a focus on surgery and prognostication, and investigates the site-specific strengths and limitations of FDG-PET/CT. Overall, the goal of treatment for NSCLC is to provide the best opportunity for long-term survival; therefore, FDG-PET/CT is expected to play an increasingly important role in deciding the appropriate treatment for such patients.

## 1. Introduction

Lung cancer is the leading cause of cancer-related deaths worldwide [1]. Non-small-cell lung cancer (NSCLC) is the most common type of lung cancer, with subtypes including adenocarcinoma, squamous cell carcinoma, and large cell carcinoma [2]. The optimal management of NSCLC depends on the histological subtype, the molecular characteristics, and the tumor stage [3].

Computed tomography (CT) is the required examination for patients with NSCLC [4]. Moreover, the histological characteristics caused by gene expression and cytokines can affect the imaging appearance either directly or indirectly [5]. 

Fluorodeoxyglucose F 18 (FDG)-positron emission tomography (PET), which enables tumoral features such as tumor metabolism or receptor expression to be visualized and quantified on a molecular level, has also become more clinically significant in the management of patients with NSCLC [6]. Both FDG-PET alone and that combined with CT (FDG-PET/CT) can combine anatomic data and metabolic information for NSCLC [7]. The anatomic data obtained using CT, including tumor size and location, is supplemented by the metabolic information obtained using FDG-PET. The standardized uptake value (SUV) is a semiquantitative measure of the tracer uptake in a region of interest that normalizes the lesion activity to the injected activity and a measure of the volume of distribution. In general, SUV was calculated as follows: tumor activity concentration/(injected dose/body weight) [8]. It is known that, in NSCLC, the expression of both hexokinase activity and glucose transporter proteins are upregulated [9]. The National Comprehensive Cancer Network (NCCN) guidelines recommend FDG-PET/CT for the evaluation of patients with NSCLC in all stages [10]. The utility of FDG-PET/CT for staging patients with NSCLC is also made reference to in the American College of Radiology Appropriateness Criteria and American College of Chest Physicians guidelines [11,12].

In a prospective, multicenter, randomized trial, conventional work-up combined with FDG-PET resulted in a 51% relative reduction in futile thoracotomy, with an overall one in five reduction in unnecessary surgery, compared with conventional work-up only [13]. In addition, in a large meta-analysis, going beyond unnecessary surgery, FDG-PET was found to have superior mediastinal staging compared with CT [14]. Overall, the combined information obtained from FDG-PET/CT hybrid imaging has been found to provide more accurate staging than either imaging modality alone [15,16,17,18,19].

FDG has become the most common tracer used in PET, owing to a number of favorable characteristics. First, FDG has to use membrane transport proteins, such as glucose transporters [20], because it does not enter tissues passively. Second, phosphorylation makes FDG incapable of export back to the extracellular space. Third, compared with adjacent tissues, the glucose transporters and hexokinase enzymes that phosphorylate and transport FDG are often overexpressed in solid tumors [7]. The high imaging contrast enhances these characteristics in many types of cancer, including NSCLC.

This review focuses on recent advances in FDG-PET imaging within the field of clinical oncology in NSCLC, with a particular emphasis on surgery and prognostication, and investigates the site-specific strengths and limitations of FDG-PET/CT.

## 2. Association between Maximum SUV (SUVmax) of the Primary Tumor in NSCLC and Pathological Findings and Prognosis

In NSCLC, bronchioloalveolar carcinoma has relatively low FDG uptake, and poorly differentiated carcinoma have been shown to have a wide range of FDG accumulation [21,22]. These findings can be explained by the varying expression levels of glucose transporter-1 and P-glycoprotein [21,22,23]. Accordingly, the World Health Organization updated its NSCLC classification criteria in 2015 [2]. FDG uptake is reported to be heterogeneous in different histological subtypes of lung adenocarcinoma, and several studies have found that histological subtypes are independent predictors of nodal metastasis, recurrence, and survival in patients with lung adenocarcinoma [2,24,25].

However, different conclusions have been reached in previous studies on the prognostic significance of SUVmax in NSCLC. Some have reported that SUVmax is not significantly related to survival in patients with resectable and advanced NSCLC [26,27,28], whereas others have claimed that in early stage NSCLC, preoperative SUVmax is a significant prognostic factor [23,25,29,30,31,32]. Kwon et al. demonstrated that the SUVmax of the primary tumor in stage I NSCLC is predictive of survival and time to recurrence [32]. An analysis of variance demonstrated that the mean SUVmax was significantly lower for adenocarcinoma than for squamous cell carcinoma and other histologies. This parameter may serve as a biomarker to guide selection of patients for adjuvant chemotherapy or other more aggressive therapies. The contrary conclusions in these studies may have been the result of patient selection, tumor stage, or the small numbers of cases. Moreover, differences in terms of the acquisition and interpretation of SUVmax can also produce inconsistent results. Therefore, the standardization of FDG-PET/CT imaging, including patient preparation, image acquisition, image reconstruction, and quantitative image analysis, is needed.

In NSCLC, false negative FDG-PET results tend to be seen in small nodules, most frequently those with a diameter < 8–10 mm, invasive mucinous adenocarcinomas with a reduced amount of cells, and carcinomas with minimally invasive adenocarcinoma and low-grade malignancy such as carcinoma in situ [33], the CT findings of which can manifest as ground-glass or part-solid ground-glass nodules. By contrast, lepidic-predominant invasive adenocarcinomas appear as mixed solid and ground-glass nodules [34]. As reported by MacMahon et al., FDG-PET/CT is indicated for the evaluation of sub-solid ground-glass nodules only if the solid component is > 8 mm [35]. Lepidic adenocarcinomas and solid-type lung cancer lesions < 8–10 mm are reportedly associated with false negative FDG-PET/CT results [36]. Among solid-type lung cancers, lesion size and histopathological findings were associated with FDG uptake [36]. In particular, it warrants attention that lesions ≤2 cm in diameter or solid-types of bronchioloalveolar carcinoma and well-differentiated carcinoma on thin-section CT images have a tendency for negative findings on FDG-PET/CT. Therefore, solid-type nodules should be observed by follow-up CT scans even though the FDG-PET findings are negative.

On the other hand, false positive FDG-PET results in NSCLC are sometimes seen in patients with inflammatory disease caused by inflammation or infection [37]. Feng et al. reported finding a 7% false positive FDG-PET/CT rate in patients with lung cancer, the causes for which included inflammatory pseudotumor (43%), tuberculoma (37%), and organizing pneumonia (6%). In a multivariate analysis, a relationship was found between the false positive rate and higher levels of interleukin-6, positive findings on an interferon-gamma release assay for tuberculosis, age < 50 years, and nondiabetic status [38].

## 3. Predictive Value of FDG-PET/CT for the Detection of Lymph Node Metastasis in Patients with NSCLC

In patients with NSCLC, lymph nodes > 1 cm in the short axis on CT are considered to show metastatic disease [39]. The nodal status of NSCLC is of particular concern to surgeons because lymph node metastasis is an important determinant of stage and prognosis [40]. However, surgeons sometimes face a discrepancy between the clinical and pathological N status. Even if tumors are diagnosed as clinical N0 (no nodal involvement), based on preoperative radiological findings, microscopic lymph node metastasis is sometimes detected by pathological evaluation after surgery [41,42,43,44,45,46,47,48,49,50,51,52,53,54].

Nodal status remains one of the most important factors in the determination of treatment strategies. As N2 (ipsilateral mediastinal or subcarinal lymphadenopathy) NSCLC is considered a systemic disease, multimodal treatment is suitable for such patients, whereas adjuvant chemotherapy is more suitable for those with N1 (ipsilateral nodes within the lung up to hilar nodes) NSCLC. For optimal treatment, nodal status needs to be diagnosed with high accuracy.

Recent studies have reported finding pathological lymph node metastasis in 6–34% of clinical N0 lung cancer cases (Table 1) [42,43,45,46,47,48,49,50,51,52,53,54,55]. If unexpected N2 metastasis is detected during an operation, lung resection will be justified to the extent that no disease will be left behind [56]. However, if N2 metastasis is detected during the preoperative evaluation, the patient becomes a candidate for multimodal treatment. Because N0 and N2 diseases should have different treatment strategies, the frequency of unexpected N2 lesions should be reduced.

**Figure 1:**
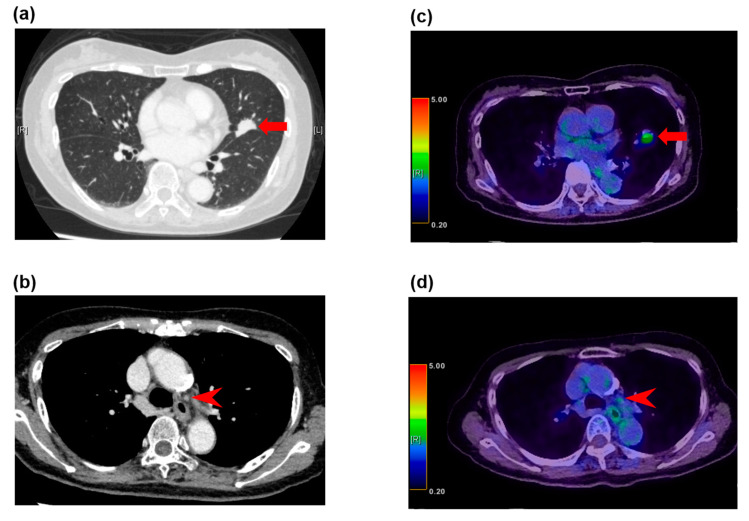
False negative fluorodeoxyglucose F 18 positron emission tomography (FDG-PET) findings in normal-sized lymph nodes in a 79-year-old woman with adenocarcinoma. (**a**) The computed tomography (CT) image shows a left upper lobe tumor (arrow), (**b**) and nonenlarged station 5 lymph nodes (arrowhead). (**c**) The FDG-PET/CT image shows FDG accumulation with a maximum standardized uptake value (SUV) of 3.3 (arrow) in the adenocarcinoma in the left upper lobe, (**d**) and no FDG accumulation in the normal-sized station 5 lymph nodes (arrowhead). These lymph nodes demonstrated metastatic disease at histopathologic examination of the specimen from surgical biopsy.

The reported ranges for the sensitivity and specificity of CT for the detection of lymph node metastasis are 51–64% and 74–86%, respectively [57]. FDG-PET/CT has been shown to have a sensitivity of 58–94% and a specificity of 76–96% for the detection of mediastinal lymph node metastasis [58]. The low sensitivity indicates a high possibility of false negative results, which may be due to low FDG uptake in a low-volume malignancy or in a malignancy with a low metabolic rate [59] (Figure 1). Therefore, surgeons cannot conclude that enlarged nodes on CT but negative findings on FDG-PET/CT are truly negative.

According to Wang et al., who carried out a meta-analysis of 10 studies using either FDG-PET/CT or a visual combination of FDG-PET and CT, the negative predictive values for mediastinal lymph node metastases were 94% and 89% in T1 and T2 tumors, respectively [60]. Therefore, invasive mediastinal staging may be omitted for patients with T1 tumors; however, it remains necessary for patients with higher-stage tumors, even if the FDG-PET/CT of the mediastinal lymph nodes is negative [61,62]. The NCCN guidelines recommend pathological and optional pathological mediastinal lymph node evaluation before resection for all stage II and solid tumors < 1 cm or purely nonsolid tumors < 3 cm with no diseased nodes identified on FDG-PET or CT [10,63].

**Figure 2:**
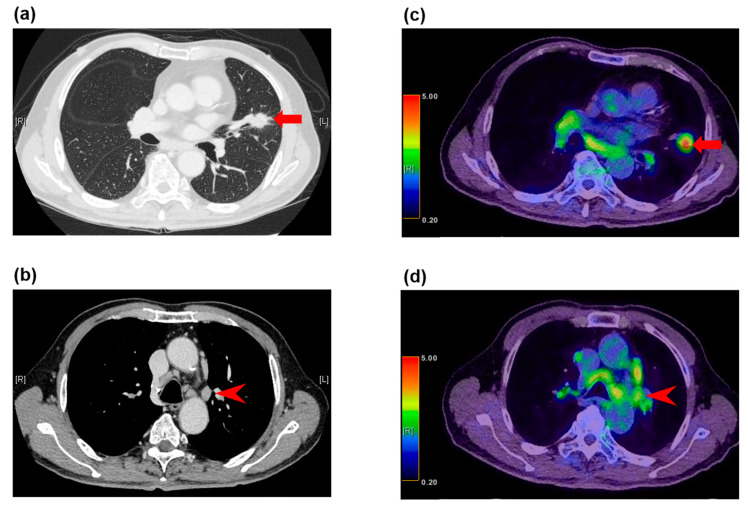
False positive FDG-PET/CT findings in enlarged lymph nodes in a 78-year-old man with squamous cell carcinoma. (**a**) The CT image shows a left upper lobe tumor (arrow), (**b**) and enlarged station 5 lymph nodes (arrowhead). (**c**) The FDG-PET/CT image shows FDG accumulation with a maximum SUV of 6.9 (arrow) in the squamous cell carcinoma in the left upper lobe, (**d**) and FDG accumulation with a maximum SUV of 3.9 in the enlarged station 5 lymph nodes (arrowhead). These lymph nodes demonstrated granulomatous inflammation at histopathologic examination of the specimen from surgical biopsy.

Regarding false positive lymph node metastasis in NSCLC staging with FDG-PET/CT, age > 65 years and non-adenocarcinoma histology have been found to be independent factors for false positive hilar and mediastinal lymph nodes [64]. In addition, nodal involvement with inflammatory lesions, such as sarcoidosis, and with granulomatous infections, such as tuberculosis, can result in false positive findings on FDG-PET, and may be related to the presence of silicosis, emphysema, or interstitial pneumonitis [65,66] (Figure 2).

Based on these findings, patients observed to have enlarged mediastinal lymph nodes on CT, regardless of whether the FDG-PET/CT findings are positive, may be candidates for invasive mediastinal staging [61,67]. Similarly, if no enlarged lymph nodes are found on CT, but the FDG-PET/CT findings in the mediastinal nodes are positive, invasive mediastinal staging is needed. Ghosh et al. reported that CT and FDG-PET/CT may both be falsely negative (CT: 20–25%, FDG-PET/CT: 24–83%) in patients with a central tumor [68]. Therefore, invasive mediastinal staging is especially recommended for patients with central and higher-stage tumors (i.e., evidence of clinical N1 disease).

## 4. Role of FDG-PET/CT in NSCLC Staging

The significance of the SUVmax on FDG-PET remains controversial [26,27,30,31]. The management of NSCLC depends on the stage, and numerous studies have reported that FDG-PET/CT is highly accurate for tumor–node–metastasis staging in patients with NSCLC [10,69]. FDG-PET/CT may be used in patients with locally advanced NSCLC suitable for treatment with curative intent to identify unsuspected metastases, which can reduce the frequency of futile thoracotomies. Progression-free and overall survival rates have been shown to be significantly worse in upstaged disease using FDG-PET/CT [70]. In NSCLC, cells show altered metabolism and utilize excess glucose as an energy source, and histological factors are modulated by FDG uptake. This method leads to gaining a better understanding of the molecular biology of NSCLC.

Based on the results of a prospective multicenter trial, when FDG-PET/CT was carried out, management strategies changed in approximately 72% of lung cancer cases [71]. In addition, according to the results of a meta-analysis of 56 studies conducted to evaluate the diagnostic value of FDG-PET/CT in NSCLC, the pooled sensitivities and specificities of FDG-PET/CT for identifying mediastinal lymph node metastasis were 72% and 91%, respectively. Moreover, FDG-PET/CT is the first-line modality for evaluating extracranial metastases in patients with NSCLC.

Distant metastases are known to occur in about 11–36% of patients with NSCLC, with the adrenal glands, liver, brain, bones, and abdominal lymph nodes comprising the most common metastatic sites [72]. The pooled sensitivities and specificities of FDG-PET/CT for detecting all extrathoracic metastases have been reported to be 77% and 95%, respectively [73]. Another meta-analysis of nine studies found that FDG-PET/CT had a sensitivity, specificity, and positive predictive value of 93%, 96%, and 28.4%, respectively, and a negative predictive value of 0.08%, for detecting distant metastases [74]. The NCCN guidelines regarding imaging appropriateness recommend that FDG-PET/CT be conducted from the base of the skull to the knees (i.e., whole-body PET/CT) for the evaluation of patients with NSCLC in all stages [10,37]. According to these guidelines, FDG-PET/CT findings confirmed to be positive for distant disease require histological or another type of radiologic confirmation, as does FDG uptake in mediastinal lymph nodes. In patients with advanced stage tumors, to identify abnormal areas that would confer the highest stage, the NCCN guidelines recommend FDG-PET before diagnostic biopsy, as well as FDG-PET/CT for the evaluation of incidentally detected lung nodules measuring >8 mm. A standardized uptake value greater than that of the baseline mediastinal blood pool is considered a positive result on FDG-PET [37,75]. Patients with a single extrathoracic metastatic lesion in a single organ (e.g., the brain, liver, bones, distant lymph nodes, skin, peritoneum, adrenal glands) have been reported to have better survival than patients with multiple extrathoracic lesions, and therefore may be candidates for local ablative therapy or surgical resection. However, in surgical candidates with a single atypical lesion, pathological confirmation is needed [76]. By contrast, the NCCN guidelines do not recommend routine bone scintigraphy for NSCLC staging.

## 5. Correlation between FDG-PET/CT and Tumor Immunometabolic Phenotypes in NSCLC

A paradigm shift has occurred in the management of NSCLC as a result of recent success in the treatment of locally advanced and metastatic NSCLC with immunotherapy [77,78,79,80]. Some studies have reported finding inverse correlations between tumor glycolytic metabolism and/or FDG uptake and tumor infiltration by effector immune cells [81,82,83]. Mitchell et al. reported identifying an association between elevated FDG retention and highly glycolytic metabolism, the expression of enhanced programmed death-ligand 1 (PD-L1), and an immunosuppressive phenotype in surgically resected NSCLCs [84]. They found that each FDG-PET parameter (SUVmax, SUVtotal, SUVmean, Total lesion glycolysis) was positively correlated with tumor expression of glycolysis-related genes. Elevated FDG SUVmax was more discriminatory of glycolysis-associated changes in tumor immune phenotypes than other FDG-PET parameters. Increased SUVmax was associated with multiple immune factors characteristic of an immunosuppressive and poorly immune infiltrated tumor microenvironment, including elevated PD-L1 expression, reduced CD57+ cell density, and increased T cell exhaustion gene signature. Elevated SUVmax identified immune-related transcriptomic signatures that were associated with enhanced tumor glycolytic gene expression and poor clinical outcomes. Their findings suggest that in patients with resectable NSCLC, FDG-PET could serve as a noninvasive clinical indicator of tumor metabolic and immune phenotypes. In addition, Faubert et al. provided direct evidence that the uptake of tumor cell-autonomous lactate, but not glucose, is a major contributor to central metabolism in human NSCLCs with high FDG-PET uptake and aggressive oncological behavior [85]. Further studies are needed to uncover more details about the contributions of immune and cancer cells to the intratumoral metabolic phenotype. Therefore, additional investigations in larger cohorts are needed to characterize fully the immunometabolic differences between NSCLC histologic subtypes, disease stages, tumor genomic features, and previous therapies, as well as the effect of these differences on the noninvasive assessment of tumor phenotypes based on FDG uptake.

## 6. FDG-PET/Magnetic Resonance Imaging (MRI)

FDG-PET/MRI technology, which can provide useful information regarding metabolic activity and tumor cellularity simultaneously, has become increasingly available. FDG-PET/MRI is a hybrid imaging modality that offers high quality morphological and functional imaging and reduces radiation exposure in comparison to FDG-PET/CT at the same time. FDG-PET/MRI is expected to be superior to FDG-PET/CT due to the superior detectability of pleural and mediastinal involvement with MRI and due to high sensitivity for the detection of brain, liver and bone metastases [86,87]. Thus, FDG-PET/MRI may be advantageous compared to FDG-PET/CT when assessing advanced tumors of the chest, both with regard to local staging accuracy and radiation dose management. For prognostic evaluation as well as tumor response assessment, the combination of metabolic information depicted by FDG-PET and functional MR imaging biomarkers could excel currently used imaging methods [88,89]. A number of studies have recently described FDG-PET/MRI as having an equivalently high diagnostic performance in staging of NSCLC [90,91,92]. Schaarschmidt et al. reported that despite the variability of FDG-PET/CT and FDG-PET/MRI in tumor, node, metastasis (TNM) staging, both modalities lead to comparable therapeutic decisions in NSCLC patients. Hence, FDG-PET/MRI can be considered a possible alternative to FDG-PET/CT for clinical NSCLC staging. These studies suggest that multidimensional functional imaging using FDG-PET/MRI is a promising method for evaluating the activity of the intratumoral environment. Still, the introduction of FDG-PET/MRI in clinical practice is hindered by several reasons. Apart from the obvious advantages of FDG-PET/CT such as the high availability and widespread experience of radiologists and nuclear medicine physicians or the shorter acquisition time if nonoptimized FDG-PET/MRI protocols are used, the sensitivity of FDG-PET/MRI for the detection of pulmonary nodules is still a matter of ongoing debate. A validation cohort in a larger population is needed to confirm these findings and establish the utility of FDG-PET/MRI.

## 7. FDG-PET/CT Radiomics and Radiogenomics

Radiomics is quantitative extraction of imaging features from medical scans. It is believed that radiomics has the potential to improve on traditional, manual interpretation by detecting features and patterns that otherwise would go unnoticed to the human eye [93,94]. By leveraging on large datasets and artificial intelligence techniques, radiomics could help predict the type of disease, survival, and response to therapy [95,96]. There are also a number of logistic advantages in this approach, such as providing nearly real-time results and not requiring any invasive procedure for the patient [97]. The overall objective of radiomics is to build classification and/or regression models based on some quantitative features extracted from the imaging data. The typical workflow in radiomics is rather independent of the underlying disease and consists of six sequential steps (acquisition, pre-processing, segmentation, feature extraction, post-processing, data analysis) [98]. Many recent studies have consistently emphasized the potential advantages of FDG-PET/CT radiomics in lung cancer [99]. The results available in the literature are undoubtedly promising, but they also need to be considered with care. The lack of reproducibility, for instance, is a well-known problem in radiomics, and is mostly a consequence of the absence of standardized methods and settings in all the steps of the workflow [98,100]. A number of studies also suffer from serious limitations at the validation level, among them improper statistical analysis and/or the absence of an independent validation dataset to confirm the results. This may easily lead to biased discovery rates and inflation of type-I errors, as correctly pointed out in [101]. As of now, the evidence of the superiority of radiomics beyond standard imaging analysis tools including SUV, kinetic data, etc. is yet to be confirmed. The availability of large, possibly multi-center, image datasets is crucial for the development and validation of radiomics methods. On the other hand, for NSCLC patients, radiomics studies have shown that several CT and FDG-PET/CT image features can predict mutation status. Ninatti et al. reported the current state of the art of imaging-derived biomarkers predictive of genetic alterations in NSCLC by using a systematic literature review [102]. CT and FDG-PET imaging-derived radiomic features, convolutional neural networks (CNN)-based approaches, FDG-PET parameters, and visual qualitative CT features were tested for the prediction of actionable mutations. Most of the published studies were focused on epidermal growth factor receptor (EGFR) alterations, which are the most commonly encountered actionable mutations in clinical practice, being present in 40–50% and 10–20% of NSCLC patients of Asian and non-Asian ethnicity, respectively [103,104,105]. The imaging-based predictive models were able to predict EGFR status, with performances ranging from poor (area under the curve (AUC) = 0.6 to 0.7, *n* = 5) to acceptable (AUC = 0.7 to 0.8, *n* = 11), excellent (AUC = 0.8 to 0.9, *n* = 18), and outstanding (AUC > 0.90, *n* = 1) in the validation set. However, the AUC of a model is not itself informative, since many other significant items, each contributing for a predetermined rate, account for the reliability of a study. Positive outcomes were also reported for the prediction of other molecular alterations, including anaplastic lymphoma kinase (ALK) rearrangement and ALK/proto-oncogene 1 (ROS1)/rearranged during transfection (RET) fusions. However, very few studies have been published with this aim, and more advanced image analyses are thus needed to confirm these preliminary results. The predictive potential of FDG-PET parameters and visual qualitative features was investigated. Zhang et al. found that a lower peak standardized uptake value (SUVpeak) was associated with EGFR mutations, while spiculation, the absence of emphysema, pleural indentation and the subsolid nodule were the semantic CT features most commonly associated with EGFR mutations [106]. However, there are no standardized definitions for visual qualitative features, and this may affect the reproducibility of results. Radiomics provides objective, repeatable and quantitative assessments. On the other hand, the possibility of analyzing images with “intelligent” methods (e.g., unsupervised), and the development of strategies to address the “black-box” and accountability issues make CNN-based approaches even more attractive in the field of medical imaging [107]. Reported results suggest that combining different methods for image biomarker extraction may help to improve the predictive performances of the models and be a winning strategy towards their implementation into clinical practice. Particularly useful were the combinations of (1) CT and FDG-PET radiomic features and FDG-PET parameters; (2) CT radiomic features and visual qualitative CT features; and (3) CT radiomic features and CNN-based approaches. The potential of combined models, therefore, needs to be investigated further with future studies. Moreover, the importance of adding clinical features to improve the performance of imaging-based predictive models must be underlined. For example, most of the included studies reported a statistically significant association of the female sex and non-smoking status with EGFR mutation. These findings were consistent with large-scale molecular epidemiological investigations that were done in patients affected by NSCLC [105,108]. Nonetheless, they did not find common, reliable radiomic features among the studies. This finding may be related to the different tools applied for feature calculation and different approaches to data analysis. Despite the promising results in terms of predictive performance, image-based models suffering from methodological bias require further validation before replacing traditional molecular pathology testing.

## 8. Conclusions

This review summarizes the recent advances in FDG-PET imaging within the field of clinical oncology in NSCLC. In terms of an overall strategy, the goal of NSCLC treatment is to provide the best opportunity for long-term survival. In that sense, FDG-PET/CT plays a very important role in deciding the appropriate treatment for patients with NSCLC. Additional multicenter prospective studies with larger cohorts and randomized controlled trials with comparator arms are needed to guide clinical management of patients with NSCLC.

## Figures and Tables

**Table 1:** Representative studies on the incidence of pathological nodal metastasis in clinical N0 lung cancer.

Author	Year	Histology	cStage	Total Number of Patients	Number of pN1/2 Patients (%)
Okada M et al. [46]	2011	adenocarcinoma	IA	502	38 (7.6)
Gomez-Caro A et al. [45]	2012	NSCLC	I	153	52 (34.0)
Koike T et al. [42]	2012	NSCLC	IA	894	67 (7.5) for pN2
Takenaka T et al. [47]	2012	NSCLC	IA	94	9 (9.6)
Zhang Y et al. [49]	2012	NSCLC	IA	530	89 (16.8) for pN2
Cho S et al. [43]	2013	NSCLC	I	770	149 (19.4)
Miyasaka Y et al. [48]	2013	NSCLC	IA-IIIA (N0)	265	51 (20.1)
Bao F et al. [51]	2014	NSCLC	T1aN0M0	315	51 (16.2)
Shiono S et al. [52]	2014	lung cancer	IA	315	20 (6.3)
Ye B et al. [53]	2014	adenocarcinoma	T1aN0M0	273	18 (6.6)
Tsutani Y et al. [50]	2015	squamous cell carcinoma	IA	100	12 (12.0)
Wang L et al. [54]	2015	peripheral lung cancer	IA	292	28 (9.6) for pN1
Kaseda K et al. [55]	2016	NSCLC	I	246	31 (12.6)

Non-small-cell lung cancer (NSCLC).
